# Giant heat transfer in the crossover regime between conduction and radiation

**DOI:** 10.1038/ncomms14475

**Published:** 2017-02-15

**Authors:** Konstantin Kloppstech, Nils Könne, Svend-Age Biehs, Alejandro W. Rodriguez, Ludwig Worbes, David Hellmann, Achim Kittel

**Affiliations:** 1 Institute of Physics, Carl von Ossietzky University of Oldenburg, D-26111 Oldenburg, Germany; 2 Department of Electrical Engineering, Princeton University, Princeton, New Jersey 08544, USA

## Abstract

Heat is transferred by radiation between two well-separated bodies at temperatures of finite difference in vacuum. At large distances the heat transfer can be described by black body radiation, at shorter distances evanescent modes start to contribute, and at separations comparable to inter-atomic spacing the transition to heat conduction should take place. We report on quantitative measurements of the near-field mediated heat flux between a gold coated near-field scanning thermal microscope tip and a planar gold sample at nanometre distances of 0.2–7 nm. We find an extraordinary large heat flux which is more than five orders of magnitude larger than black body radiation and four orders of magnitude larger than the values predicted by conventional theory of fluctuational electrodynamics. Different theories of phonon tunnelling are not able to describe the observations in a satisfactory way. The findings demand modified or even new models of heat transfer across vacuum gaps at nanometre distances.

## Abstract (web summary)

Kloppstech *et al*. report experimental observations of the heat transfer between a gold tip and an atomically flat gold sample in the 0.2–7 nm regime. The observed flux rates are four orders of magnitude larger than expected from theory, suggesting the possibility of additional heat transfer mechanisms.

The radiative heat flux between two massive bodies held at different temperatures increases drastically when the distance *d* between them becomes smaller than the dominant thermal wavelength *λ*_th_, roughly 10 μm at room temperature. Consequently, the heat flux can be enhanced by many orders of magnitude compared with the heat transfer exchanged between two black bodies coupled through the far-field. This super-Planckian effect can be attributed to the additional contribution of evanescent waves such as frustrated total internal reflection modes, surface phonon polaritons, surface plasmons, or hyperbolic modes^1^,^2^. The heat flux enhancement in the near-field regime has been verified by a number of recent experiments^3^,^4^,^5^,^6^,^7^,^8^,^9^. So far, the measured data has enjoyed good agreement with theoretical models of macroscopic heat transfer^3^,^4^,^5^,^6^,^7^,^8^,^9^,^10^, suggesting that super-Planckian radiation is a well-understood phenomenon and shifting the focus of current experiments/theory to the design of practical and more efficient near-field transmitters^11^,^12^.

However, commonly used theoretical models of heat transfer are based on Rytov’s theory of macroscopic fluctuational electrodynamics^13^, which cannot fully describe heat exchange at distances down to a few nanometres. In particular, such a theory does not account for the cross-over from near field to contact, in which case the objects are separated by atomic distances and heat flux is mediated by conductive transfer. Several recent theoretical works have studied this cross-over by including effects like tunnelling of acoustic phonons^14^,^15^,^16^ and quantum effects due to the overlap of the electronic wave functions^17^, showing that the radiative heat flux can be further enhanced by several orders of magnitude at distances of a few nanometres or even on the sub-nanometre level. The above-mentioned experiments have confirmed the predictions of the conventional macroscopic theory^3^,^4^,^5^,^6^,^7^,^8^,^9^ as they probe the near-field at much larger distances. Up to now, only one indirect measurement conducted by Altfeder *et al*.^18^ is allegedly backing the theoretical considerations on phonon tunnelling using inelastic scanning tunnelling microscopy. In contrast, compared with previous experiments our set-up is the only one which can directly probe heat fluxes for distances, precisely at the interface of radiative and conductive transport.

In this work, we report experimental observations of the heat transfer between a gold tip and an atomically flat gold sample in the ultra-small distance regime of 0.2–7 nm gained with a near-field scanning thermal microscope (NSThM). We exploit exact numerical methods based on fluctuational electrodynamics to determine the theoretical heat flux for the configuration used in the experimental set-up and show that the experimentally observed flux rates are four orders of magnitude larger than expected. Our findings suggest the possibility of additional heat conduction mechanisms and demand a modified theory capable of describing heat transport in the crossover regime between conduction and radiation.

## Results

### Experimental set-up

**Figure 1:**
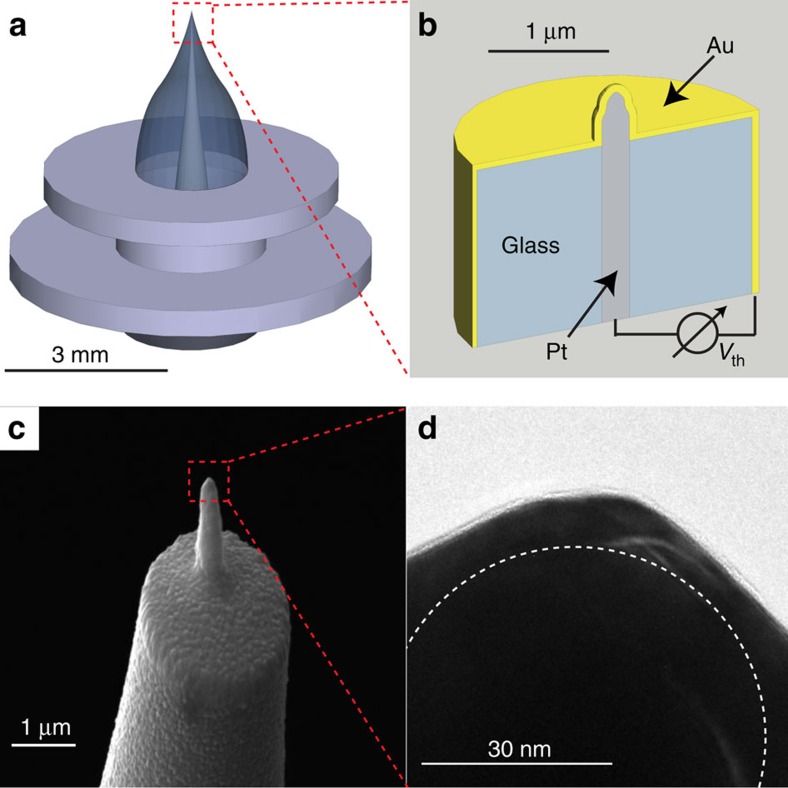
NSThM probe. (**a**) Sketch of the probe in its Omicron-type tip holder. (**b**) Schematic cross-section of the sensing end of the probe. A thermocouple is formed where the gold coating separates from the platinum core. (**c**) Scanning electron microscope micrograph of a typical NSThM probe. (**d**) Transmission electron microscope image—more precisely, the shadow because the tip is too thick to be transparent for electrons—of the tip of a typical NSThM probe indicating a radius of curvature of about 30 nm (dashed semicircle). Here the axis of rotational symmetry lies in the vertical direction.

Our experiment is performed with a custom-built NSThM under highly controlled conditions in ultra-high vacuum with a typical working pressure of 10^−10^ mbar. The set-up is based on a commercial scanning tunnelling microscope (STM). As depicted in Fig. 1a,b, the home-made STM probes consist of a platinum wire, molten into a glass capillary, pulled sharp with a pipette puller and are then coated with 100 nm of Au by means of e-beam evaporation *ex situ*. At the point where the Au film separates from the Pt-core, a thermocouple is formed. This probe design allows for local heat flux measurements in addition to its STM ability^19^,^20^. A heat flux coupled into the tip apex drains towards the back side of the tip holder causing a temperature difference between them which, finally, is generating a thermoelectric voltage *V*_th_. A scanning electron microscope image of such a probe is depicted in Fig. 1c. The protruding part of the probe is typically about 1–2 μm in length and 300–700 nm in diameter (at the base). The radius *r* of the tip apex is typically about 30 nm (ref. 21), as shown in the transmission electron microscope micrograph in Fig. 1d.

Our probes are able to detect heat fluxes down to 4 nW and heat conductances down to 24 pW K^−1^ at 50 Hz bandwidth. As we will see below, this sensitivity of the probe is not sufficient to measure radiative heat fluxes predicted by fluctuational electrodynamics. Concerning the heat fluxes, we achieve a lateral resolution of 6 nm when a temperature difference Δ*T* between probe and sample is applied^19^,^20^. The topographic information can be measured at the same time using the STM ability of our probe which features atomic resolution (Supplementary Note 1 and Supplementary Fig. 2 in Supplementary Note 2).

### Experimental results

**Figure 2:**
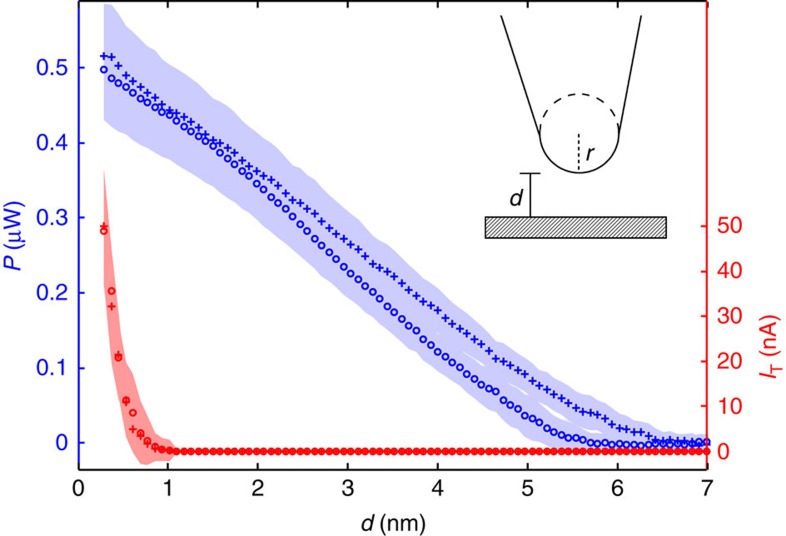
Gap-dependent heat flux and tunnelling current. Measured average heat flux power *P* (blue curves with respect to the axis on the left-hand side) and tunnelling current *I*_T_ (red curves with respect to the axis on the right-hand side) as a function of distance *d* for approaching (circles) and retracting (crosses) direction together. The sample given by a 200 nm gold-film on a mica substrate is cooled down to 120 K, whereas the temperature of the probe is held at ambient temperature so that Δ*T*=160 K. The shaded areas quantify the uncertainties: In case of the tunnelling current the uncertainty is given by its standard deviation, whereas the relative error of the heat flux measurement is calculated via Gaussian error calculus for each distance step. The certainty of the value for the distance *d*=0 nm is limited by the certainty of the value for the work function for gold^37^. From this we estimate a relative error in *d*=0 nm of Δ*d*=90 pm. Inset: sketch of the probe and the sample.

The measured change of the probe-sample heat current Δ*P* in the distance regime of 0.2–7 nm is ∼0.5 μW as shown in Fig. 2. This corresponds to a heat transfer coefficient *h*_nf_ through the vacuum gap by near-field interactions of


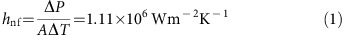


when using Δ*P*=0.5 W and assuming a disk-shaped effective heat flux area *A* of the tip with *r*=30 nm and a temperature difference of Δ*T*=160 K, since *T*_probe_=280 K and *T*_sample_=120 K. In contrast, the heat transfer coefficient between two black bodies at the same temperatures can be estimated to be


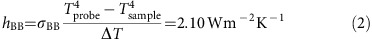


using the Stefan–Boltzmann constant *σ*_BB_=5.67 × 10^−8^ Wm^−2^K^−1^. Hence, the measured heat transfer coefficient of the vacuum gap is about 5 × 10^5^ times larger than the black body value. Thus, our NSThM technique yields by far the largest heat flux level compared with other near-field experiments^3^,^4^,^5^,^6^,^7^,^8^,^9^. So far, these have measured heat fluxes up to ∼100 times the black body value^7^, albeit at much larger distances. As we show below, this value is four orders of magnitude larger than that obtained using conventional macroscopic fluctuational electrodynamics. However, theoretical models based on phonon tunnelling can predict such large values. Furthermore, we find at close distances up to *d*=2 nm an almost linear decay of the heat flux, which means that we can exclude algebraic decays of the form *d*^−*n*^ with *n*≥1, but we cannot exclude exponential decays.

We want to emphasize that the measured heat transfer cannot be caused by Joule heating from tunnelling electrons. The maximum power of Joule heating by the tunnelling electrons can be estimated to be 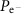
=*VI*_T_=30 nW (*V*=600 mV and *I*_T_=50 nA) which is about six per cent of the maximum measured heat flux. Furthermore, Fig. 2 shows the typically observed exponential decay of the tunnelling current *I*_T_ at distances below *d*=1 nm. At larger distances no current is detectable anymore (below 0.5 pA). Thus, the massive heat flux and its distance dependence cannot be explained by the exchange of electrons.

## Discussion

**Figure 3:**
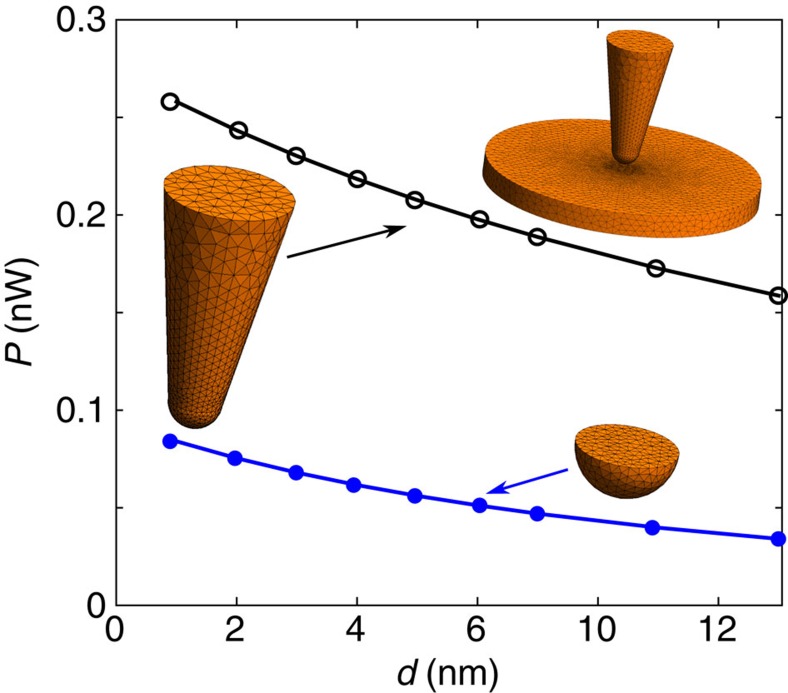
Theoretical results of the transferred heat flux. Sketch of the considered geometry and numerical results using exact numerical calculations for the spherical tip and the cone-like protruding part. The parameters of the tip are the following: the foremost part is modelled by a sphere of radius of 30 nm, the protruding conical part has a length of 300 nm with a radius at the base of 87.5 nm.

Now, we want to compare commonly discussed theoretical models with our experimental data. Let us first stick to the conventional macroscopic theory: in Fig. 3 we show exact numerical results for the radiative heat flux using a boundary-element method in order to model the geometry of our probe^22^,^23^. Obviously, the values for the heat flux are between 0.2 nW(*d*=5 nm) and 0.25 nW(*d*=0.5 nm) which corresponds to heat transfer coefficients *h*_nf_ of about 440–550 Wm^−2^K^−1^. Therefore, the change of the probe-sample heat current as predicted by fluctuational electrodynamics is only 110 Wm^−2^K^−1^, whereas the measured value is 1.1 × 10^6^ Wm^−2^K^−1^. It is interesting that the distance dependence of the heat flux found in Fig. 3 is similar to the measured distance dependence, but the heat flux level is obviously four orders of magnitude too small. Thus, the curvature of the tip cannot account for the discrepancy between the experimental data and the theory.

It might be argued that non-local response of the permittivity should be taken into account^24^,^25^,^26^,^27^. Since non-local effects cannot be included easily in the exact numerical scheme, we have also modelled our sensor using the so-called proximity approximation (PA), which can be applied in the near-field regime when the distance between two objects is much smaller than their curvature^28^,^29^,^30^,^31^,^32^. Note, that this approximation has successfully been applied in all experiments using a spherical probe^4^,^5^,^8^,^9^. In our case, however, we obtain results for the whole probe which show a similar distance dependence as the exact results in Fig. 3, but which overestimate the contribution of the tip and lead to errors on the order of 400% (see Supplementary Note 3). Similar deviations of the PA and exact results were already observed in a sphere-plate geometry (see ref. 29) and here can be traced back to the fact that the foremost part of the tip is extremely small compared with *λ*_th_ so that it acts more like a dipole than a macroscopic sphere. Nonetheless, one can still use the PA to estimate the heat flux in our set-up, keeping in mind that it overestimates the heat flux level. Now, within this approximation we have included non-local effects using the Lindhard–Mermin model^24^ (see Supplementary Note 4), and we find that while these non-local effects increase the heat flux, as expected^24^,^26^, they turn out to be relatively weak for the considered distances. On the other hand, the inclusion of surface roughness^30^,^31^,^33^ and the interplay of radiation and conduction^34^ will decrease the predicted heat flux level shown in Fig. 3 which will cancel the slight effect of enhancement due to non-local effects. Hence, we find that the conventional macroscopic model of heat transfer greatly underestimates the heat flux found in our experiments. We note and emphasize that the above calculations in Fig. 3 employ no approximation and fully account for flux mediated by surface plasmon polaritons, though these tend to be negligible for gold at room temperature^24^. A possible explanation for the enhanced heat transfer could be the phenomenon of phonon tunnelling. However, at the moment no theoretical model of phonon tunnelling is capable of describing all of the features observed in our experiment. In particular, some of these models predict an algebraic decay of the heat flux that is not in accordance with our data. A more detailed discussion of phonon tunnelling can be found in the Supplementary Note 5.

The giant heat flux observed in this geometry begs the question: at what distances is near-field radiative heat transfer as described by macroscopic fluctuational electrodynamics valid? Unfortunately, the sharp tip required to perform well defined measurements at short distances and the high sensitivity needed to detect small power rates at large distances make such a measurement (requiring observations from nanometre to hundreds of nanometre gaps) in this set-up extremely challenging. One potential avenue for future work is to employ a transition-edge sensor, which works only at temperatures of a few Kelvin, meaning that the system must be cooled down to these temperatures.

In conclusion, we have measured the near-field mediated heat flux between a gold coated near-field scanning microscope tip and a gold sample at distances of a few nanometres. The measured values for the heat flux are four orders of magnitude larger than predicted by fluctuational electrodynamics and five orders of magnitude larger than the black body value. The observed heat transfer is highly localized with a characteristic length scale of a few nanometres (see Supplementary Note 6). Therefore, it is predestined for applications like heat assisted magnetic recording with a pit size of about 25 nm^2^ which corresponds to a storage density of 25 Tb in^−2^. A comparison with current models of phonon tunnelling shows that they can explain why the heat flux can be much larger than predicted by fluctuational electrodynamics, but these models typically predict an algebraic decay of the heat flux which is in contradiction to the measured decay. Given the current lack of appropriate theoretical models that can span this range of distances and geometries, the question of whether the measured heat flux can be explained by phonon or photon tunnelling, or whether there is yet another unknown mechanism at play, remains open. We hope, however, that this work along with previous results on a related experiment^15^, provide the basis and motivation for further theoretical exploration of heat transfer mechanisms in this crossover regime where both radiative and conductive effects can coexist and are greatly affected by geometry.

## Methods

### Characterization and calibration of the probe

We use 1*ω*-measuring techniques along with an adapted hot-wire method. In a first step our probe is characterized *in situ* just before the actual measurement to obtain the ratio *ε* between the heat flux through the probe and the hereby generated thermoelectric voltage *V*_th_. To this end, we use 1*ω*-measuring techniques along with an adapted hot-wire method. The details of this calibration method can be found in ref. 35. For the NSThM probe used in our measurements we find a calibration factor of *ε*=0.43 μW per μV. The heat flux through the probe is given by *P*=*εV*_th_. The purely near-field contribution to the heat transfer is detected by subtracting the heat flux at larger distances (typically a few tens of nanometres) realizing that the near-field effect has fallen below the detection limit at these distances. Our calibration method enables us to measure absolute heat fluxes between the probe and the sample with a relative uncertainty of about 14% (details of the error analysis leading to this uncertainty can be found in ref. 35).

### Characterization of the sample

The sample used in our measurement consists of a 200 nm Au layer deposited *ex situ* via e-beam evaporation on a cleaved and heated mica substrate, leading to a monocrystalline Au(111) surface. After cleaning (sputtering with Ar ions and annealing) it shows wide atomically flat areas and the common 22 × 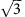 surface reconstruction (see Supplementary Note 2). In a next step, the sample is cooled down to 120 K, while the probe is held close to ambient temperature leading to a probe-sample temperature difference of Δ*T*=160 K.

### Measurement of the heat flux

Using the STM ability of our probe, first an atomically flat and clean area of about 75 × 75 nm^2^ (see Supplementary Note 2 and Supplementary Fig. 2) is approached where we then perform our measurements of the heat flux from the probe’s tip to the sample. This is done, firstly, by lifting the probe 7 nm from the tunnelling distance (tunnelling current *I*_T_=1 nA, bias voltage *V*_T_=600 mV). Then, the sample is approached stepwise at a maximum slew rate of 90 nm s^−1^ until a threshold of the tunnelling current of 50 nA is reached, which corresponds to a sample-probe distance *d* of about 0.2 nm. While approaching the surface the thermoelectric voltage is acquired after a settling time and an integration time of 20 ms, both, using distance steps of about 0.08 nm. Then the probe is retracted again and the heat flux is measured using the same measurement procedure. Finally, the tip is brought back to its original tunnelling distance. Averaged data of the heat flux for approaching and retracting measurements are shown in Fig. 2. Note, that only those measurements are taken into account where the difference in the piezo stroke at the beginning and at the end of the measuring cycle is less than 50 pm. This is done to avoid drift artifacts, spoiling the averaging procedure of 100 measurements for each direction. We emphasize that here *d*=0 nm corresponds to the inter-atomic distance in bulk gold. Therefore, the value *d*=0 nm denotes the distance where the electrical conductance of a single-Au atom (*I*_T_=46.5 μA; 600 mV) is reached in the STM. We estimate a relative error in separation between tip and sample surface *d* of Δ*d*=90 pm. This precision can only be achieved because of the atomically flat surface of the sample and the small tip radius, a well known fact in the context of Casimir forces^36^, which is a unique feature of our heat transfer measurements on the nanoscale (for details see Supplementary Notes 7 and 8).

### Numerical modelling

The theoretical predictions of the heat flux in Fig. 3 were obtained via a recently developed fluctuating-surface current formulation of heat transfer that is applicable to arbitrary geometries and has been validated against well-established results in more conventional geometries, including planar and spherical objects^22^,^23^. Our results were obtained using a free numerical implementation of this method based on the boundary-element method, which discretizes the object surfaces (the scattering unknowns) using localized Rao–Wilton–Glisson basis functions. The model geometry follows the same parameters as the experiment, with material parameters described via the standard Drude model of Au (see Supplementary Note 9), except that the cone height is capped at 300 nm and the planar sample is taken to be a cylinder with a finite radius of 600 nm and a thickness of 50 nm. We find that larger cone heights and sample diameters have a negligible impact on the overall heat transfer, as does the contribution of the planar base (see Supplementary Note 3). Figure 3 shows a schematic of the discretized geometry, which is chosen to yield converged results with respect to the mesh size (resolution) and geometric parameters. Comparison between the numerical and PA predictions shows that the latter greatly overestimates the impact of the sphere-tip in comparison with the conical section as well as the overall flux (see Supplementary Note 3).

### Data availability

The data that support the findings of this study are available from the corresponding author upon request.

## Additional information

**How to cite this article:** Kloppstech, K. *et al*. Giant heat transfer in the crossover regime between conduction and radiation. *Nat. Commun.* **8,** 14475 doi: 10.1038/ncomms14475 (2017).

**Publisher’s note**: Springer Nature remains neutral with regard to jurisdictional claims in published maps and institutional affiliations.

## Supplementary Material

Supplementary Information Supplementary Figures and Supplementary Notes and Supplementary References
