# Publisher Correction: Giant heat transfer in the crossover regime between conduction and radiation

**DOI:** 10.1038/ncomms16224

**Published:** 2018-06-25

**Authors:** Konstantin Kloppstech, Nils Könne, Svend-Age Biehs, Alejandro W. Rodriguez, Ludwig Worbes, David Hellmann, Achim Kittel

Nature Communications 8: Article number: 14475; DOI: 10.1038/ncomms14475 (2017); Published: 02 15 2017, Updated: 06 25 2018

The original HTML version of this Article omitted the article number; it should have been ‘14475’. This has now been corrected in the HTML version of the Article. The PDF version was correct from the time of publication.

