# Supplementary material for: Giant heat transfer in the crossover regime between conduction and radiation
Source: Nat Commun. 2017 Feb 15;8:14475. doi: 10.1038/ncomms14475 (PMC5330847; doi:10.1038/ncomms14475)
Supplement: Supplementary Information — Supplementary Figures and Supplementary Notes and Supplementary References [file ncomms14475-s1.pdf]

Supplementary Note 1. Heat conduction in different parts of the near-field scanning thermal microscope.

All details concerning the probe and its features are published in Fleischmann-Wischnath et al.[1] In a brief summary: The probe consists of a Pt wire molten into a glass capillary, which is then pulled sharp with a pipette puller and coated with gold (see Fig. 1 of the article). This coaxial design can be represented by a parallel circuit of thermal resistances of three different parts: The first is the gold film, the second is the Pt wire, and the third is of course the resistance of the glass capillary. It is possible to determine the thermal resistances of the metallic parts by measuring the electric resistance and using the Wiedemann-Franz law. Next, it is possible to calculate the capillary's thermal resistance by measuring the geometry of the glass capillary and utilizing literature values for the thermal resistivity of the used glass. We have done this in the above mentioned publication[1], explaining in detail all occurring heat transfers in the probe.

The probe's back is held close to ambient temperature, while the protruding part of the tip is cooled down ($\Delta T < 1$ K) when the probe is approaching the tunneling distance to the cooled gold sample. The heat flux through the sensor tip causes a temperature drop along the thermal resistance of it. The resulting temperature difference leads to a thermopower, measured in the coaxial thermocouple included in the probe. Thus the thermoelectric voltage is directly connected to the heat flux from the probe to the sample. We have developed an *in situ* calibration method to relate the thermoelectric voltage directly to the occurring heat flux through the probe. We have used this interrelation to determine the heat resistance and the calibration factor of the sensor as it is described in detail in Kloppstech et al.[2] This method does not rely on pure calculations or estimations, but on a precise measurement routine to evaluate the exact relation between the occurring heat flux and the generated thermoelectric voltage at different heat fluxes.

The sample holder used in the experiments is a modified version of the original Omicron VT-STM sample holder (see Supplementary Figure 1). It basically consists of an oxygen free copper block in the center, which is fixed between a ceramic circuit board (No. 1 in Supplementary Figure 1 (a)) and a bottom plate made from stainless steel. The assembly is held together by four M1.2 molybdenum screws and nuts to avoid migration of conducting material in the vicinity of the ceramic circuit board during annealing processes. This board provides electrical connections to the sample in general. It is used to drain the tunneling current from a sample in standard experiments (No. 4 in Supplementary Figure 1 (a)). For the calibration procedure a Pt wire is connected employing the four-wire technique. The four-wire setup is used to measure precisely the electrical resistance without interface resistances. For example it is employed to measure the resistance of an added Pt100 resistance thermometer using these contacts. Its conducting paths consist of a gold plated copper layer.

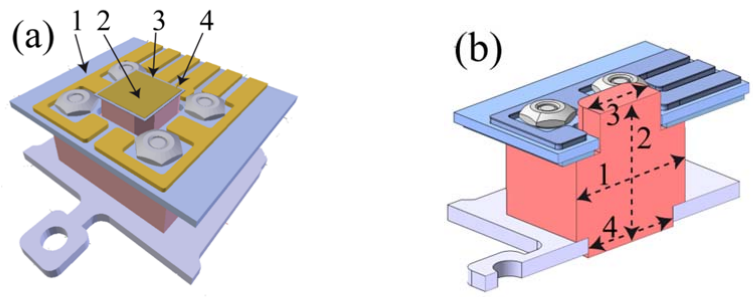

Supplementary Figure 1: **Schematic drawing of the sample holder which is a custom made design.** (a) Assembly of the sample holder (1 Ceramic circuit board, 2 Au layer, 3 mica sheet, 4 wire to drain the tunneling current). (b) Cut open representation of the sample holder (1 width of 9 mm, 2 height of 9 mm, 3 sample platform of 5 x 5 mm² area, 4 coupling area 7 mm in diameter). The holder consists of a stainless steel base plate, a copper block in the center, and a ceramic circuit board, which are held together by four molybdenum screws.

The center copper block has a width of 9 mm (Arrow 1 in Supplementary Figure 1(b)) and a height of about 9 mm (Arrow 2 in Supplementary Figure 1(b)). The bottom of this copper block is coupled via a copper braid to the cold finger of a He flow cryostat. For convenience we operate our cryostat with liquid nitrogen most of the time. The sample holder is pressed to a gold plated copper piece which is directly soldered to the copper braid. The copper piece's temperature can be controlled by means of a Lakeshore temperature controller which drives a heater incorporated into it (stability better than 0.1 K).

The copper piece is coupled to the copper block of the sample holder over an area with diameter of 7 mm (Arrow 4 in Supplementary Figure 1(b)) by pressing them together with a screw. On the top of this block (Arrow 3 in Supplementary Figure 1(b)) our gold sample is glued with a thin layer of conductive silver paint and contacted with a 50 µm gold wire (No. 4 in Supplementary Figure 1(a)) for tunneling current measurement over a 5 x 5 mm² area.

One can assume that the thermal coupling between the copper block and the gold coated mica to be homogeneous and evenly distributed over the whole 5 x 5 mm² area on top of the copper block. The sample is thermally contacted to our cryostat's cool finger at the bottom area. That means that the whole block is cooled down to a temperature of about 120 K. The ceramic circuit board is cooled on its back side, being thermally coupled to the copper block of the sample holder. The board is electrically contacted by means of six zero force contact fingers which are pressed to each individual pad by a lever mechanism. The lever mechanism is coupled to components at room temperature leading to a heat flux through the contacts into the ceramic circuit board. This heat flux together with the heat radiation of the 300 K surrounding are the reason for the relatively high sample temperature of about 120 K.

The copper block with its geometrical configuration has a thermal resistance of about 0.4 K/W, when cooled to 120 K. The silver paint (we assume here a 10 µm thick layer, covering the 5 x 5 mm² top area entirely) has a thermal resistance of about 0.1 K/W, while the mica plate has an estimated thermal resistance of about 1 K/W. These thermal resistances are seven orders of magnitude smaller than that of the vacuum gap at tunneling distance which is about $5.3 \cdot 10^7$ K/W according to Kloppstech et al.[2] Therefore, one can neglect the distortion of the local surface temperature of the gold film, caused by the warmer probe, even if the tip is in tunneling distance to the sample surface. This results in a well-defined temperature difference across the tunneling gap.

Supplementary Note 2. Samples preparation and surface cleanliness.

Before performing the measurements the samples and the tip are carefully cleaned under ultrahigh vacuum conditions. Between sample cleaning and the actual measurements the sample and the tips are always kept under ultrahigh vacuum conditions, therefore, we avoid surface contamination as common in surface science. The gold sample consists of an approximately 25 μm thin mica sheet (No. 3 in Supplementary Figure 1(a)), on which a gold layer (No. 2 in Supplementary Figure 1(a)) of 200 nm thickness is deposited via e-beam evaporation. Before starting the actual measurement we perform a few cleaning cycles of the samples. One cleaning cycle consists of a sputter phase and a subsequent annealing phase in order to achieve widely atomic flat areas. The sputter phase is purely physical sputtering with Argon ions (5 – 15 min.) but we tried different reactive sputtering procedures too (with O_2 and H_2). During the annealing phase the sample is heated together with the sample holder to above 500 °C for about half an hour in ultrahigh vacuum[3,4]. The tip is cleaned by electron bombardment (for details see [5,6] or Argon ion sputtering under ultrahigh vacuum).

Before each measurement the surface quality is checked by taking an image of the surface topography and the heat transfer between sample and tip. The measurements of the distance dependence are only performed in the center of extended flat surface areas of at least 100 nm x 100 nm. This area is by far large enough taken into account that the observed heat transfer decays on a length scale of about 6 nm. Supplementary Figure 2(a) shows an example of a typical surface area used for the measurements. To illustrate the surface quality and the power of the NSThM, Supplementary Figure 2(b) shows an image on the atomic scale which exhibits the small corrugation of the gold surface due to the atoms. From our lateral measurements we know that single atomic layer steps do influence the heat transfer in a very minor way (see Supplementary Figure 3(b) and Worbes et al.[3,4]).

(a)

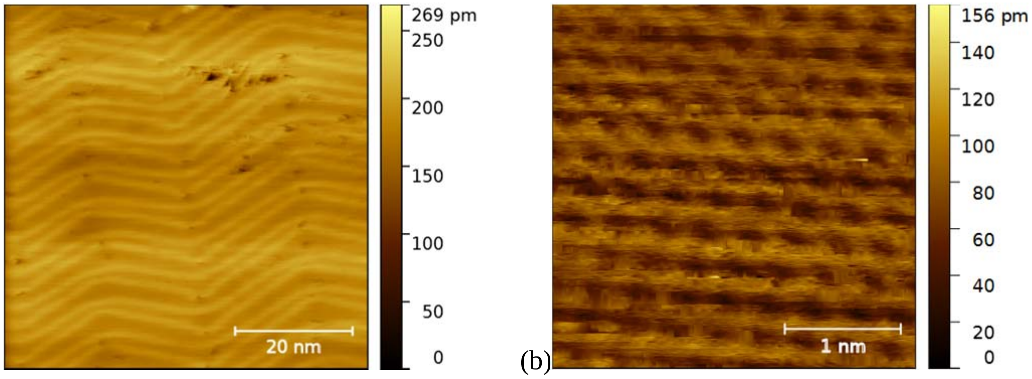

Supplementary Figure 2: **Topographic STM image of the gold surface.** (a) An example of a flat part sample surface is shown. The surface exhibits the herring bone reconstruction which is typically observed for gold (111) surfaces. (b) Topographic image of the surface with nearly atomic resolution by means of the NSThM tip. The reduced resolution in comparison to an STM is a consequence of the relatively large curvature of the NSThM tip.

The scan feature of the NSThM is very important to examine the quality of the surface. This concerns not only the surface roughness but also the cleanliness of the surface. Sometimes one can observe a situation exemplarily illustrated in Supplementary Figure 3. The topographic image shows in some regions structures which appear to be reduced in height (see Supplementary Figure 3(a)). This is presumably caused by a surface contamination with a material possessing a larger work function than gold. This changes drastically the heat transfer in this area (see Supplementary Figure 3(b)). Thus it is of elementary importance to avoid these kinds of contaminations and check the sample's cleanliness each time before starting measurement of quantitative heat transfer. In such cases we perform further

cleaning cycles to ensure clean sample surfaces. Therefrom we have learned that it is essential to clean the samples within ultrahigh vacuum and keep it there without exposing it to any higher pressure until the actual measurement is performed to avoid any contamination by adsorption processes from the gas phase.

Single atomic layer steps of the gold surface have a minor influence on the heat transfer but only if it is right under the center of the tip apex. This is indicated by the fine lines in the Supplementary Figure 3(b) at each step of a single atomic layer.

(a) (b)

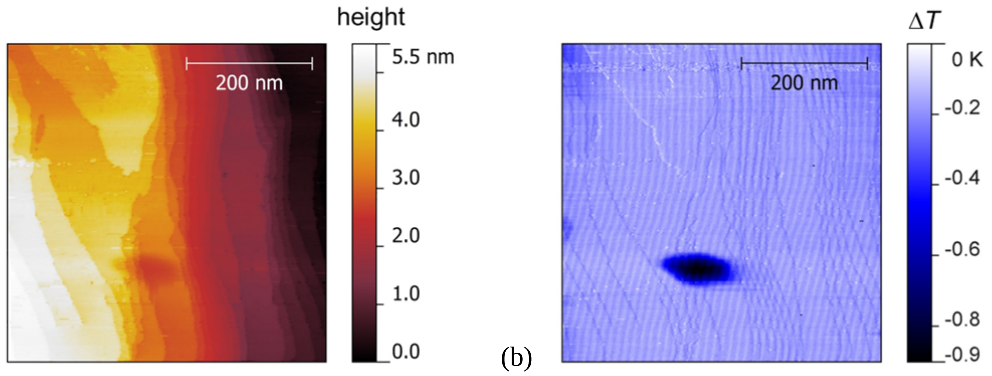

Supplementary Figure 3: **Comparison between topographic and heat flux image.** (a) Topographic image of the surface of a gold (111) surfaces. It can be seen that this part of the surface exhibits many steps of atomic layers. In the center of the image's lower third one observes a roundly shaped shadow which pretends an area of reduced height. (b) Image of the lateral heat transfer distribution recorded at the same time as taking the topographic image. The shadow region of part (a) shows an enhanced heat transfer whereas the steps of atomic layers do not possess a specific signature. They can be hardly seen in the heat transfer image. Such kind of samples can be used to crosscheck the calibration of the *z*-piezo displacement over a larger distance.

In our previous work[7] we have used a much less filigree sensor with a larger tip radius fabricated with an entirely different process. The control equipment for the STM essential for the separation control was only able to retract the tip from tunnel separation but not to approach it again reliably (Omicron Scala). We had to measure the heat flux by only retracting the tip. We performed an averaging process at each separation during a single retraction stroke. Furthermore, we were not able to anneal the sample in our UHV chamber. After publication we developed the new sensors and switched to a new control equipment for the STM (Nanonis). With these two essential improvements we were able to perform cycles of retraction and approaching the surface again with our sensors. It turned out that there must be a surface contamination because we observed a large hysteresis (of a few nanometer up to tens of nanometer) during these cycles and sometimes an abrupt and erratic switching to zero heat transfer upon retraction of the tip. A tedious investigation turned out that the switching was depending on the retraction velocity which is again a finger print of surface contamination. This drove us to an in-situ cleaning procedure of the tip and the sample which was a challenge but it was the only way to carefully prepare flat and clean samples and clean tips without contamination showing reproducible retraction-and-approaching cycles in the heat transfer.

At the moment, there is no possibility to absolutely proof that there is no surface contamination because smallest amounts like a few atoms sitting between the tip and the surface could have an

influence on the heat transfer. We used Auger electron spectroscopy which is already sensitive to small amounts (below one percent of a monolayer) without any signature of contamination. Thereby we can exclude monolayer coverage of the surface by any contaminants. Any kind of electron microscopy is not applicable because exposing the sample to air or non HV conditions would already contaminate the sample. In addition, the exposure to the electron beam would lead to a contamination mainly by carbon from residues in the vacuum by the mechanism of electron beam-induced deposition (EBID). By the way, this is the reason why the tip cannot be imaged before the experiment by electron microscopy without cleaning it afterwards which will change the tip shape again. As discussed already the behavior of the tunnel current only points to an electrically insulation contamination.

Supplementary Note 3. Proximity approximation.

As shown recently[17,18] for distances d smaller than the tip radius of our sensor, the curvature of the sensor can be taken into account within the so-called proximity approximation (PA), which can be applied in the near-field regime when the distance between two objects is much smaller than their curvature[17,18,19,20].

We first divide the probe into its three main parts to model the heat flux: the foremost spherical part, the conical protruding, and the planar base (see Supplementary Figure 5). The mean heat flux ⟨S⟩ (W/m²) between the planar base and the planar sample is calculated with the Polder-van Hove heat-flux formula[21] and then multiplied by the circular ring area of the planar base with inner radius of 350 nm and outer radius of 1250 nm. In order to model the spherical foremost part and the conical protruding part we use the PA. In this approximation the transferred power between the spherical part and the substrate is expressed by

$$P_{\text{sphere}} = 2\pi \int_0^r \mathrm{d}s\, s \langle S(\tilde{d}(s)) \rangle \quad (2)$$

where $\langle S \rangle$ is the heat flux between two planar materials obtained with the Polder-van Hove heat-flux formula[21] and $\tilde{d}(s) = d + r - \sqrt{r^2 - s^2}$. A similar expression

$$P_{\text{cone}} = 2\pi \int_r^R \mathrm{d}s\, s \left\langle S\left(\tilde{\tilde{d}}(s)\right)\right\rangle \quad (3)$$

applies for the contribution of the conical part, where $\tilde{\tilde{d}}(s) = d + r + L\frac{s-r}{R-r}$, where $L = 1200$ nm is the length of the protruding part and $R = 350$ nm is the radius of this conical part at its base.

In Supplementary Figure 5, the values for the heat flux using the PA are between 0.5 nW and 1 nW which corresponds to heat transfer coefficients h_{nf} of about $1.1 - 2.2 \cdot 10^3\ \mathrm{W/m^2}$K. Furthermore, approximating the probe as a perfectly sharp cone, of dimensions comparable to our experiment, results in even smaller heat fluxes in accordance to exact numerical calculations[22,23,24]. It is interesting that the distance dependence of the heat flux found in the PA in Supplementary Figure 5 is similar to the measured distance dependence, but the heat flux level is three to four orders of magnitude too small. Comparing the PA results with the results of the exact numerical method, we find that PA overestimates the heat flux in the considered geometry by a factor of roughly 4. Such deviations even in regions where the radius of curvature is much larger than the distance were also found in previous works[18,25].

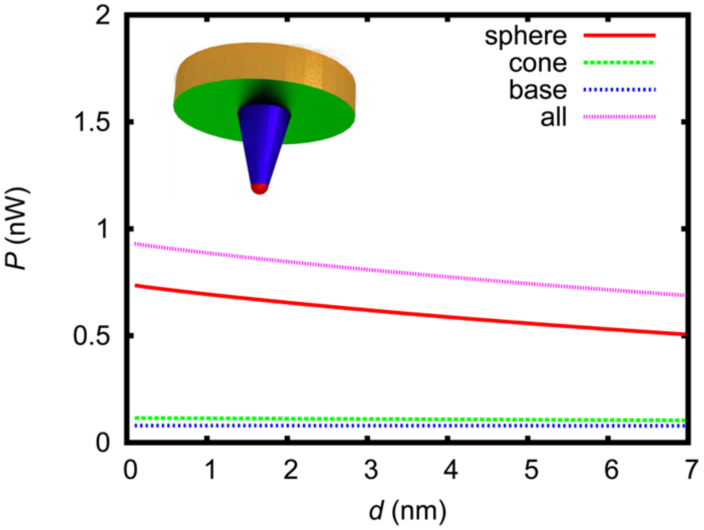

Supplementary Figure 4: **Theoretical results of the transferred heat flux.** Depicted is a sketch of the considered geometry (see inset) and numerical results using proximity approximation for the spherical tip and the cone-like protruding part and exact results for the base of the tip. We have dyed the different parts of the probe (see inset) with the same colors as used for the lines in the plots. The parameters of the tip are the following: the foremost part is modeled by a sphere of radius of 30 nm, the protruding conical part has a length of 1200 nm with a radius at the base of 350 nm and the washer shaped base itself has an outer radius of 1250 nm.

Supplementary Note 4. Nonlocal contribution to the radiative heat flux.

The heat flux between the NSThM tip and the substrate can be estimated by the heat flux between two gold half-spaces multiplied by the disk-shaped effective area A of the foremost part of the tip, which is assumed to be spherical. The numerical results using the standard expressions of the mean Poynting vector $\langle \mathrm{S} \rangle$ derived by Polder and van Hove[21] (PvH) with the optical data of gold[15] are shown in Supplementary Figure 6 for $r = 30\,\mathrm{nm}$. Obviously, the heat flux ranges from 1 nW to 2 nW, which corresponds to heat transfer coefficients h_{nf} of about $2.2 - 4.4 \cdot 10^3\ \mathrm{W/m^2K}$. More precisely, the increase in heat flux is approximately $0.5\,\mathrm{nW}$ ($h_{\mathrm{nf}} = 1.1 \cdot 10^3\ \mathrm{W/m^2K}$) in the range of our measurement, i.e., our experimental results are three orders of magnitude larger than the result of this model. Note, that the contribution of the non-protruding part of the tip, will result in a nearly constant offset of less than 0.1 nW, which is not measured, since only heat flux differences are measured.

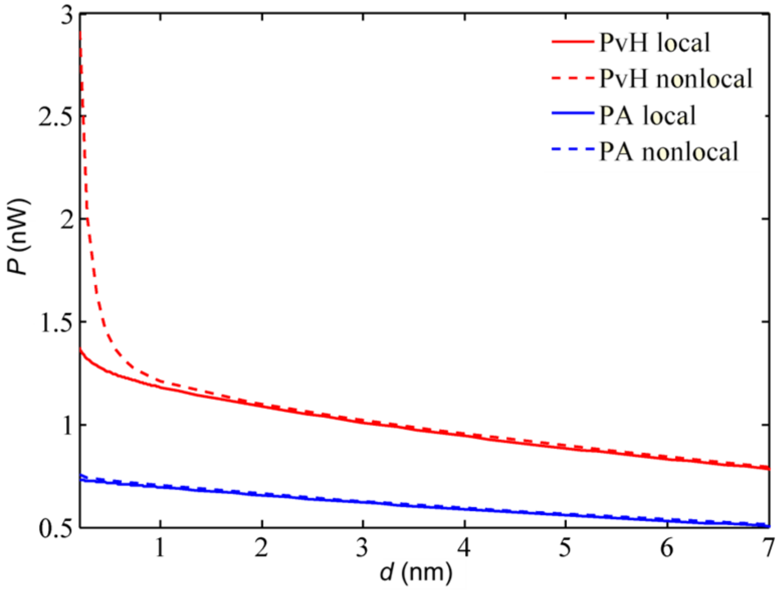

Supplementary Figure 5: **Heat flux with local and nonlocal theory.** Calculated heat flux P between two gold surfaces held at $T_1 = 280$ K and $T_2 = 120$ K for local (solid lines) and nonlocal (dashed lines) approaches. On the one hand, the power is determined by calculation of the heat flux ⟨S⟩ (mean Poynting vector) between two planar gold surfaces multiplied by the effective cross section of the foremost part of the tip $A = \pi r^2$ using $r = 30$ nm (labeled with PvH). On the other hand, the curvature is taken into account by the PA, again assuming a spherical tip with radius $r = 30$ nm.

Supplementary Note 5. Discussion of models of phonon tunneling.

We consider a number of currently accepted models of phonon (conductive) transfer and argue that they too cannot explain the above-mentioned enhancement:

(A) Prunnila and Meltaus[26] have studied the tunneling of acoustic phonons between piezoelectric materials. They report an approximate $1/d^3$ distance dependence. Making the same estimation for the effective tip area A as above we find that for piezoelectric materials studied by Prunnila and Meltaus[26] a power transfer of about 13 nW could be expected for $r = 30$ nm at $d = 1$ nm ($h_{\mathrm{nf}} = 2.9 \cdot 10^4$ W/m^{2}K). This value is too small to explain our data and the distance dependence does not agree with the measured one. But this is not surprising, since in our experiment we are not using piezoelectric materials but metals, meaning that this theory cannot be applied directly.

(B) Another approach is given by Sellan et al.[27] who consider phonon tunneling between two silicon half spaces through a vacuum gap using lattice dynamics calculations. The authors report a heat flux by phonon tunneling (resulting in a heat transfer coefficient h_{nf} of $5.3 \cdot 10^8$ W/m^{2}K) which is five orders of magnitude larger than the conventional radiative heat flux at $d = 1$ nm. Although the reported enhancement is about two orders of magnitude larger than ours, this effect is only observable at distances smaller than 0.2 nm, above which this theory coincides well with macroscopic fluctuational electrodynamics. Hence, phonon tunneling within this model also cannot explain our enhancement which occurs at distances up to 5 nm. Furthermore, the calculations were only done for Si using a specific Stillinger-Weber potential usually used in bulk material. It should be mentioned that using another atomistic simulation method, it was shown very recently that for polar materials like

SiC phonon tunneling only slightly increases the heat flux with respect to Rytov's theory in the distance regime between 0.2 nm and 1 nm[28].

(C) The approach of Mahan[29] based on image potentials considers heat tunneling between a metal and alkali halides so that it is again not directly applicable to our experiment. The theory predicts a $1/d$ dependence with very large heat fluxes by phonon tunneling even for several nanometers. At distances of 2 – 3 nm, the heat flux drops by a factor of 10 compared to the boundary Kapitza conductance between metals and alkali halide, which yields a heat-transfer coefficient on the order of $h_{\mathrm{nf}} = 10 - 100 \cdot 10^6$ W/m^{2}K. Hence, while such a model does predict heat fluxes of similar magnitudes as those obtained here at certain separations, we find that it cannot explain the distance dependence observed in our measurements. Furthermore, this model cannot be directly applied to the case of two metal surfaces.

(D) Yet another but very general description for phonon tunneling was proposed by Budaev and Bogy[30] based on a very elementary classical oscillator model for describing lattice vibrations. The authors find in their model that the heat flux mediated by phonon tunneling scales like $1/d^8$. For Si they find that the heat flux by phonon tunneling equals the conventional radiative heat flux at $d =$ 5 nm ($h_{\mathrm{nf}} \approx 10^5$ W/m^{2}K) and dominates the heat flux for smaller distances. This model obviously already gives different results than predicted by the much more elaborate method (B). Furthermore, the predicted $1/d^8$ distance dependence does not agree with our data.

Supplementary Note 6. Lateral resolution of the heat transfer.

By scanning across the edge of a NaCl island[4] one can experimentally determine the lateral decay length of the heat transfer. Supplementary Figure 4 shows a line scan of the heat transfer across the edge of a NaCl island as indicated by a red line in the inset of the figure. We use a 20%/80% criterion for the step height to determine the lateral decay length of the heat transfer. We observe a decay length of about 6 nm. This decay length defines the resolution limit for a heat transfer image. One can expect that the contributions laterally further away from the tip than about 6 nm do not contribute in a significant way to the heat transfer observed here.

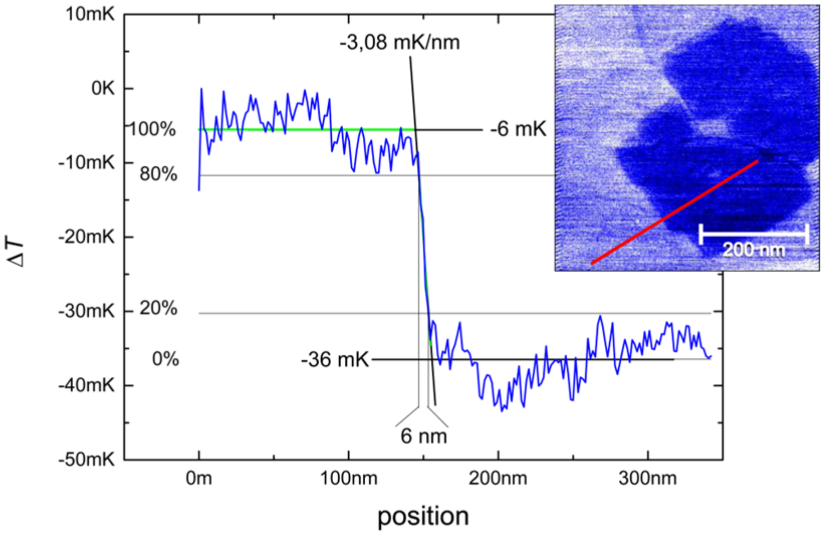

Supplementary Figure 6: **Lateral resolution of the near field scanning thermal microscope.** The image shows a line scan of the measured heat transfer across the edge of a NaCl single atomic layer island. The inset of the figure shows an image of the heat transfer which was recorded while scanning across the samples surface at tunneling distance recording the topography. The added red line to the image shows the position and the direction of the line scan. The temperature of the tip is decreased above the NaCl island due to an enhanced heat transfer which causes a stronger cooling of the tip.

Supplementary Note 7. Calibration of the piezo actuators.

As usual in STM microscopy the scanner of the microscope was calibrated in *xy*-direction by means of imaging a (7x7)-reconstruction of a silicon (111) surface and in *z*-direction by comparing the observed height of a gold mono-layer with literature values. The smallest distance between the probe and the surface of (0.2 ±0.09) nm was estimated by using the tunneling resistance. Its uncertainty is due to the general uncertainties of the tunneling matrix elements. For a calibration in the range of a few nanometer we use samples with a high density of monolayer steps like the one shown in Supplementary Figure 3 to crosscheck the piezo coefficient in this range. For the scale of a few hundred nanometers we are using commercially available calibration standards.

Supplementary Note 8. Precisely defined separation of tip and surface.

The investigation of the heat transfer at small distances is only possible if the zero separation can be defined properly and if the surface roughness is by far smaller than the resolution of the position accuracy. For all scanning probe microscopy methods one uses short range interactions to define the separation between tip and sample surface. It holds the shorter the interaction the higher the resolution. If one uses a tip with a large curvature radius and a finite surface roughness it becomes impossible to predict at what point the tip is "touching" the surface and, therefore, to define zero separation with high precision. This problem is well known in the scientific community investigating the Casimir force experimentally[8]. There are many groups using extended cross section area, i.e. "tips" with a relatively large curvature radius, to increase the total heat flux[9,10,11,12,13]. They do it for a good reason because the resolution of the measurement of the heat flux will increase linearly with the cross section

area. Most of the groups specify their smallest separation above the rms value of their sample surface and sphere used as a second surface. Rousseau et al.[11] investigated their sphere by electron microscopy and concluded from the surface roughness a uncertainty of a few tens of nanometers. They end up introducing the separation as a fit parameter explicitly because of that. Broer et al.[8] find in their analysis that the uncertainty in separation is by a factor three to five larger than the rms of the surface roughness. Only one group was allegedly able to specify the heat transfer down to separations which were only a fraction of the assumed rms value of the surface roughness[13].

We have chosen a different route to measure the heat flux at short distances. We stay with tip radius of about 30 nm as small as possible and have to sacrifice resolution of the heat transfer. This is only meaningful if we are sure that our sample surface is flat, i.e. the roughness is as small as possible. We can check that by using the STM functionality of our setup. We can specify our uncertainty in separation by $\Delta d = 90$ pm including the fact that we have to model the tunnel gap with parameters to specify zero separation. These parameters cannot be determined independently. Here we have to rely on accepted values from literature which vary. The uncertainty is small enough to specify the separation properly. For comparison in [13] the tip radius in case of the Au-Au measurement is 450 nm with an rms value of the surface roughness of 10 nm. This results in an uncertainty in the definition of zero separation at least of the same size which is two orders of magnitudes larger than that of our experiment ($\Delta d = 90$ pm). We perform our measurements starting from the smallest separation which is more than twice of our uncertainty at about 0.2 nm. At smaller separations the offset error would lead to unreliable measurements.

Supplementary Note 9. Drude parameters used in numerical simulation of the fluctuating-surface current formulation.

For the optical data we employ the Drude model

$$\varepsilon_{\mathrm{Au}} = \varepsilon_\infty - \frac{\omega_{\mathrm{p}}^2}{\omega(\omega + \mathrm{i}\tau^{-1})} \quad (1)$$

with the parameters $\varepsilon_\infty = 9.84$, $\varepsilon_p = 1.36 \cdot 10^{16}\ \mathrm{rad/s}$, $\tau = 1 \cdot 10^{-14}$ s. These parameters result from a fit[14] to the optical data of Christy and Johnson[15] which are well accepted in the plasmonic community. Nonetheless, it should be kept in mind that for each sample the parameters can slightly differ[16] which will result in slightly different results for the numerical values of the heat flux.

In general the Drude model describes the situation in the regime of infrared radiation well. Only at higher energies above 2 eV deviations can be observed. Because of the thermal excitation of the system considered here, low energy modes are excited and, therefore, the model captures well the physics involved.

Supplementary References

1. U. Fleischmann-Wischnath, J. Welker, and A. Kittel, The near-field scanning thermal microscope, Rev. Sci. Instrum. **79**, 073708 (2008).
2. K. Kloppstech, N. Könne, L. Worbes, D. Hellmann, and A. Kittel, Dancing the tight rope on the nanoscale—Calibrating a heat flux sensor of a scanning thermal microscope, Rev. Sci. Instrum. **86**, 114902 (2015).
3. L. Worbes, D. Hellmann, and A. Kittel, Enhanced Near-Field Heat Flow of a Monolayer Dielectric Island, Phys. Rev. Lett. **110**, 134302 (2013).

4. L. Worbes, Untersuchung des Nahfeld-Wärmeübertrags an adsorbatbelegten und nanostrukturierten Oberflächen, PhD Thesis, University of Oldenburg (2014).
5. D. Hellmann, L. Worbes, and A. Kittel, Compact device for cleaning scanner-mounted scanning tunneling microscope tips using electron bombardment, Rev. Sci. Instrum. **82**, 083701 (2011).
6. D. Hellmann, L. Worbes, K. Kloppstech, N. Könne, and A. Kittel, Investigation of the time evolution of STM-tip temperature during electron bombardment, J. Vac. Sci. Technol. A **31**, 031602 (2013).
7. A. Kittel, W. Müller-Hirsch, J. Parisi, S.-A. Biehs, D. Reddig, and M. Holthaus, Near-Field Heat Transfer in a Scanning Thermal Microscope, Phys. Rev. Lett. **95**, 224301 (2005).
8. W. Broer, G. Palasantzas, J. Knoester, and V. B. Svetovoy, Significance of the Casimir force and surface roughness for actuation dynamics of MEMS, Phys. Rev. B **85**, 155410 (2012).
9. L. Hu, A. Narayanaswamy, X. Chen, and G. Chen, Near-field thermal radiation between two closely spaced glass plates exceeding Planck's blackbody radiation law, Appl. Phys. Lett. **92**, 133106 (2008).
10. S. Shen, A. Narayanaswamy, and G. Chen, Surface phonon polaritons mediated energy transfer between nanoscale gaps, Nano Lett. **9**, 2909-2913 (2009).
11. E. Rousseau, A. Siria, G. Jourdan, S. Volz, F. Comin, J. Chevrier and J.-J. Greffet, Radiative heat transfer at the nanoscale, Nature Photonics **3**, 514-517 (2009).
12. S. Shen, A. Mavrokefalos, P. Sambegoro, and G. Chen, Nanoscale thermal radiation between two gold surfaces, Appl. Phys. Lett. **100**, 233114 (2012).
13. K. Kim, B. Song, V. Fernández-Hurtado, W. Lee, W. Jeong, L. Cui, D. Thompson, J. Feist, M. T. Homer Reid, F. J. García-Vidal, J. C. Cuevas, E. Meyhofer, and P. Reddy, Radiative heat transfer in the extreme near field, Nature **528**, 387–391 (2015).
14. C. Sönnichsen, Plasmons in metal nanostructures, PhD thesis (2001)
15. P. B. Johnson and R. W. Christy, Optical Constants of the Noble Metals, Phys. Rev. B **6**, 4370-4379 (1972).
16. V. B. Svetovoy, P. J. van Zwol, G. Palasantzas, J. T. M. D. Hosson, Optical properties of gold films and the Casimir force, Phys. Rev. B **77**, 035439 (2008).
17. M. Krüger, T. Emig, and M. Kardar, Nonequilibrium Electromagnetic Fluctuations: Heat Transfer and Interactions, Phys. Rev. Lett. **106**, 210404 (2011).
18. C. Otey and S. Fan, Numerically exact calculation of electromagnetic heat transfer between a dielectric sphere and plate, Phys. Rev. B **84**, 245431 (2011).
19. S.-A. Biehs and J.-J. Greffet, Near-field heat transfer between a nanoparticle and a rough surface, Phys. Rev. B **81**, 245414 (2010).
20. S.-A. Biehs and J.-J. Greffet, Influence of roughness on near-field heat transfer between two plates, Phys. Rev. B **82**, 245410 (2010).
21. D. Polder and M. Van Hove, Theory of Radiative Heat Transfer between Closely Spaced Bodies, Phys. Rev. B **4**, 3303-3314 (1971).
22. A. P. McCauley, F. S. S. Rosa, A. W. Rodriguez, J. D. Joannopoulos, D. A. R. Dalvit, and S. G. Johnson, Structural anisotropy and orientation-induced Casimir repulsion in fluids, Phys. Rev. A **83**, 052503 (2011).
23. A. W. Rodriguez, M. T. Homer Reid, and S. G. Johnson, Fluctuating-surface-current formulation of radiative heat transfer for arbitrary geometries, Phys. Rev. B **86**, 220302(R) (2012).
24. A. W. Rodriguez, M. T. Homer Reid, and S. G. Johnson, Fluctuating-surface-current formulation of radiative heat transfer: Theory and applications, Phys. Rev. B **88**, 054305 (2013).
25. K. Sasihithlu and A. Narayanaswamy, Proximity effects in radiative heat transfer, Phys. Rev. B **83**, 161406(R) (2011).
26. M. Prunnila and J. Meltaus, Acoustic Phonon Tunneling and Heat Transport due to Evanescent Electric Fields, Phys. Rev. Lett. **105**, 125501 (2010).
27. D. P. Sellan, E. S. Landry, K. Sasihithlu, A. Narayanaswamy, A. J. H. McGaughey, and C. H. Amon, Phonon transport across a vacuum gap, Phys. Rev. B **85**, 024118, (2012).
28. V. Chiloyan, J. Garg, K. Esfarjani and G. Chen, Transition from near-field thermal radiation to phonon heat conduction at sub-nanometre gaps, Nat. Comm. **6**, 6755 (2015).
29. G. D. Mahan, The tunneling of heat, Appl. Phys. Lett. **98**, 132106 (2011).
30. B. V. Budaev and D. B. Bogy, On the role of acoustic waves (phonons) in equilibrium heat exchange across a vacuum gap, Appl. Phys. Lett. **99**, 053109 (2011).
